# Quality of Life of Pregnant Women With Medical Disorders in Ibadan, Nigeria

**DOI:** 10.7759/cureus.72133

**Published:** 2024-10-22

**Authors:** Oluwasomidoyin Bello, Olaolu Oni, Dorcas Ajekola, Margaret Ogunkuade

**Affiliations:** 1 Obstetrics and Gynaecology, University of Ibadan, Ibadan, NGA; 2 Nursing, University of Ibadan, Ibadan, NGA; 3 Obstetrics and Gynaecology, University College Hospital, Ibadan, NGA

**Keywords:** chronic medical illnesses, medical illnesses, pregnant women, quality of life, who quality of life brief version (whoqol-bref)

## Abstract

Background

Pregnant women’s psychological and physical states may be greatly impacted by pregnancy-related changes, especially those with medical disorders. This study aimed to determine the quality of life (QoL) of pregnant women with medical disorder(s), associated factors, and the most affected domain.

Methodology

A cross-sectional study was conducted among 337 antenatal attendees with at least one medical disorder in a tertiary hospital using a self-administered questionnaire. The World Health Organization Quality of Life Brief Version standardized questionnaire was used to assess the QoL of pregnant women across various domains. Data were analyzed using SPSS version 27 (IBM Corp., Armonk, NY, USA). Univariate and bivariate analyses were used to examine the differences in QoL scores across various domains and maternal variables with the level of statistical significance set at p-values <0.05.

Results

The mean age of the participants was 32.43 (±4.74) years and the mean overall QoL score was 85.04 ± 9.61 with high health satisfaction at 84.10 ± 10.74. The psychological domain was most affected with a QoL of 62.78 ± 9.26. Hypertensive disorders were most prevalent 77 (23.0%). Those who had a single medical disorder had a better QoL compared to those with multiple medical disorders, 85.15 ± 9.67 vs. 84.06 ± 9.11, respectively. Educational status (p = 0.04) and occupation (p < 0.01) were significantly associated with overall QoL. Gestational age was associated with the women’s QoL in the physical health domain (p = 0.01).

Conclusions

The QoL of pregnant women with medical disorder(s) was good; however, there is a need to improve their socioeconomic and psychological support. Educating a girl child might improve her QoL during pregnancy.

## Introduction

Pregnancy is a natural and important phase of a woman’s life when her body gradually undergoes certain physiological and anatomical changes [1]. This period is characterized by important physical and emotional changes that can affect the quality of life (QoL) of pregnant women and the health of the mother and the infant [2]. Although becoming pregnant is a wonderful and desired experience, it can also bring along a lot of difficulties, discomfort, and even mood swings or sadness owing to different physical and physiological changes [3]. The hormonal, emotional, psychological, and physical factors unique to pregnancy change the physical, mental, social, and overall health dimensions of pregnant women during pregnancy and easily affect and threaten their QoL [4].

The QoL of pregnant women and its related factors are assessed across four domains, namely, physical, psychological, social, and environmental. The physical domain is influenced by changes in the body due to pregnancy and an enlarged uterus, leading to common issues such as back pain, heartburn, and sleep disturbances. The psychological domain reflects the mental state of pregnant women, often marked by feelings of worthlessness, mood swings, low self-confidence, and reliance on others for routine daily tasks. Environmental factors examine how well the woman’s needs are met by her environment, including access to appropriate clothing and facilities that accommodate her new status. Lastly, the social domain focuses on how pregnancy impacts a woman’s social interactions, such as work, social gatherings, and self-care [5,6]. These issues often worsen over time, significantly affecting pregnant women’s health status [6]. These changes may even be more difficult for expectant women with underlying medical illnesses such as asthma, peptic ulcer diseases (PUD), cardiac diseases, and hypertensive disorders to cope with and may also be an additional load on the underfunded healthcare system in developing countries. For instance, having to keep a lot of medical appointments and taking a lot of medications might be necessary [7]. QoL decreases generally in pregnancy and varies across the trimester, especially in physical and environmental domains, but is majorly affected by the presence of underlying ailments or comorbidities [8]. Therefore, a pregnant woman’s likelihood of negative outcomes for both the mother and child is enhanced if she has one or more underlying diseases [9]. Hence, ensuring a good QoL for pregnant women is one of the most significant aspects of prenatal care [10]. QoL is widely recognized as a vital indicator for assessing the physical and mental health of pregnant women, providing valuable insights into their overall well-being. Studies have shown that a decrease in physical activity is associated with higher rates of depression, anxiety, and fatigue among pregnant women compared to their non-pregnant peers, which directly impacts their physical and mental health [10,11]. Other factors from previous studies that might affect the QoL of pregnant women include age, educational status, trimester of pregnancy, monthly income, etc. [11,12].

People differ in their expectations for their health and QoL, and those who are in comparable circumstances may assess their QoL differently [12]. Numerous studies have examined pregnant women’s QoL but most concentrated solely on those with certain illnesses such as gestational diabetes, hypertension, and depression. However, there is a dearth of information in the literature about the QoL of pregnant women with other underlying disorders. The QoL of pregnant women with medical illnesses can be improved through a better understanding of their challenges.

This study assessed the QoL and associated factors among pregnant women with medical disorders to identify the most affected domain. Understanding these aspects will help clinicians offer appropriate care, treatments, and suitable environments to enhance QoL and achieve favorable fetal and maternal outcomes among pregnant women with medical disorders.

## Materials and methods

This was a cross-sectional study conducted at the antenatal clinic of University College Hospital (UCH) Ibadan, a tertiary facility in South-Western Nigeria from June to September 2022. Purposive sampling was used to select 337 pregnant women with medical disorder(s) such as hypertensive disorders, gestational diabetes, asthma, and sickle cell diseases seeking antenatal care at UCH. UCH acts as a referral center for private and other government hospitals, offering specialized care in all aspects of obstetrics and gynecology, including fetomaternal medicine. Pregnant women with medical disorder(s) are managed via a multidisciplinary approach with other specialists as needed.

The recruitment of pregnant women with medical disorder(s) into the study was based on their medical records. All antenatal clinic attendees diagnosed with medical disorder(s) were informed about the study and given the opportunity to enroll after a careful explanation of the study objectives. Written informed consent was obtained from all consenting women who met the inclusion criteria. The inclusion criteria included being 18 years and above, having one or more medical illnesses for at least three months, having a registered pregnancy, and having had two antenatal clinic visits. The exclusion criteria were multiple pregnancies because they could have exaggerated symptoms of pregnancy, especially in the physical domain, and pregnant women with congenital fetuses because the growth and the psychological attachment to pregnancy might be altered because of expected adverse outcomes.

The World Health Organization QoL-Brief Version (WHOQOL-BREF) questionnaire, a validated reliable tool for evaluating QoL in both healthy and unwell patients in different areas of health, was used [13]. This was adapted to include sociodemographic, obstetrics, and medical characteristics. The WHOQOL-BREF contains 26 questions, two regarding the overall QoL and health satisfaction, and the remaining 24 questions evaluate the following four domains: physical (seven items), psychological (six items), social relations (three items), and environment (eight items). These domains were assessed using a positive scale, i.e., the higher the score, the better the quality of life. Responses were given on a five-point Likert scale with a possible score ranging from 1 to 5. The raw scale score was converted to a scale from 0 to 100, with zero indicating the worst state of health, and 100 the best state of health. The mean score of questions in each domain was used to calculate the domain score and was finally transformed linearly to a 0 to 100 scale [13].

Data were coded, entered, and analyzed using SPSS version 27.0 (IBM Corp., Armonk, NY, USA). Medical disorder(s) were classified as either single disorder or multiple disorders (more than one medical disorder). Mean and standard deviation (SD) were used to characterize continuous variables. Frequencies and percentages were used for the demographic variables. One-way analysis of variance (F) and the independent t-test were used to determine the association between the QoL domains and the independent variables (maternal sociodemographic, obstetric, and medical characteristics) and the dependent variable (QoL). The level of significance was set at p-values <0.05.

Ethical approval was obtained from the Joint University of Ibadan and University College Hospital Ethics Review Committee (approval number: UI/EC/21/035).

## Results

A total of 337 pregnant women with medical disorders participated in this study. Most participants were aged 32 years and below (189, 56.1%) and had a higher level of education (tertiary) (263, 78.0%). The majority (239, 70.9%) of the women were Christians and were mostly from the Yoruba tribe (271, 80.4%). Most of the pregnant women were living in rented apartments (309, 91.7%) and more than half (202, 59.9%) earned more than 50,000 naira monthly ($30). The majority of the women had two children (127, 37.7%) and were in their second trimester of pregnancy (212, 62.9%). About four-fifths (305, 90.5%) had only one medical disorder while 32 (9.5%) had multiple medical disorders (Table 1).

**Table 1:** Pregnant women’s sociodemographic and obstetric characteristics. SD: standard deviation

Variable	Frequency, n (%)
Age (years)	
≤32	189 (56.1)
>32	148 (43.1)
Mean ± SD	32.43 ± 4.74
Educational level	
None	3 (0.9)
Primary	1 (0.3)
Secondary	70 (20.8)
Tertiary	263 (78.0)
Religion	
Christianity	239 (70.9)
Islam	95 (28.2)
Traditional	3 (0.9)
Ethnicity	
Yoruba	271 (80.4)
Igbo	60 (17.4)
Hausa	5 (1.5)
Others	1 (0.3)
Marital status	
Single	14 (4.2)
Married	320 (95.0)
Separated/Divorce	3 (0.9)
Occupation	
Waged employment	94 (28.0)
Unemployed	25 (7.1)
Self-employed	218 (64.9)
Accommodation	
Self-owned	28 (8.3)
Rented	309 (91.7)
Monthly income (Naira)	
≤10,000	8 (2.4)
>10,000–≤20,000	94 (27.9)
>20,000–≤50,000	33 (9.8)
>50,000	202 (59.9)
Parity	
0	79 (23.4)
1	118 (35.0)
2	127 (37.7)
3	13 (3.9)
Mean ± SD	2.22 ± 0.85
Trimester (gestational age)	
First	7 (2.1)
Second	212 (62.9)
Third	118 (35.0)
Mean ± SD	2.33 ± 0.51
Medical disorder(s)	
Single	305 (90.5)
Multiple	32 (9.5)

Among the 305 participants with a single medical disorder, gastrointestinal tract diseases (peptic ulcer and hepatitis B) were the most common (131, 42.9%), followed by cardiovascular diseases, mainly hypertensive disorders (80, 26.2%). Neurological and psychiatric conditions were the least prevalent at seven (2.3%) and one (0.3%), respectively. Meanwhile, asthma and PUD were noted in about a quarter of the women who presented with multiple medical disorders, followed by five (15.6%) who presented with hypertensive disorders and PUD (Table 2).

**Table 2:** Percentage distribution of single and multiple medical disorders in pregnant women. GDM: gestational diabetes mellitus; PUD: peptic ulcer disease; HIV: human immunodeficiency disease

Variable	Frequency (305)	Percent
Body system affected		
Cardiovascular	80	26.2
Respiratory	32	10.5
Endocrine	21	6.9
Neurological	7	2.3
Hematological	21	6.9
Immunological	12	3.9
Gastrointestinal	131	42.9
Psychiatry	1	0.3
Multiple medical illnesses (n = 32)		
Asthma and PUD	8	25.0
Hepatitis B and GDM	1	3.1
Hepatitis B and PUD	2	6.3
GDM and HIV	1	3.1
Hypertensive disorders and asthma	1	3.1
Hypertensive disorders and GDM	2	6.3
Hypertensive disorders and glaucoma	2	6.3
Hypertensive disorders and hepatitis B	1	3.1
Hypertensive disorders and HIV	2	6.3
Hypertensive disorders and PUD	5	15.6
Hypertensive disorders, asthma, and GDM	1	3.1
GDM and PUD	2	6.3
Seizure disorder and PUD	1	3.1
Sickle cell disease and GDM	2	6.3
Sickle cell disease and PUD	1	3.1

The participants perceived their QoL to be the best in the social (72.77 ± 9.77), environment (66.00 ± 8.45), and physical health domains (64.92 ± 9.42) and slightly low in the psychological domain (62.92 ± 9.42). However, the majority were highly satisfied with their health (84.10 ± 10.74) (Table 3).

**Table 3:** Satisfaction level of pregnant women in different domains. QoL: quality of life

Domains	Minimum (%)	Maximum (%)	Mean	Standard deviation
Physical health	35.71	96.43	64.92	9.42
Psychological	37.50	91.67	62.78	9.26
Social relations	33.33	100.00	72.77	9.77
Environment	46.88	100.00	66.00	8.45
Health satisfaction	40.00	100.00	84.10	10.74
Overall QoL	30.00	100.00	85.04	9.61

The overall QoL was slightly higher among those with a single medical disorder compared to those with multiple medical disorders, but this was lower in the environment domain. This difference was not statistically significant (p > 0.05) (Table 4).

**Table 4:** Association between QoL and medical illnesses among pregnant women. QoL: quality of life; SD: standard deviation

Variable	Illnesses		t-test	P-value
	Single	Multiple		
Overall QoL (mean ± SD)	85.15 ± 9.67	84.06 ± 9.11	0.64	0.53
Physical health	65.22 ± 9.11	62.05 ± 11.78	1.48	0.15
Psychological	62.81 ± 9.27	62.50 ± 9.29	0.18	0.86
Social relations	72.81 ± 9.83	72.40 ± 9.33	0.24	0.81
Environment	65.87 ± 8.57	67.29 ± 7.14	-1.04	0.30

There was a significant association between the women’s age and physical health domain. Those who were 32 years old and younger experienced higher QoL than those older than 32 years in the physical health domain (p < 0.01). A significant association was found between educational status and overall QoL (p = 0.04). The highest scores in the QoL domains were seen in women with post-secondary education, and the lowest in participants with primary or no education (p < 0.05). The overall QoL (p < 0.01) and QoL scores in the psychological (p < 0.01) and environment (p < 0.01) domains were significantly higher among pregnant women who were on salaried/waged employment. The overall QoL scores decreased among pregnant women in different trimesters of pregnancy, with those in the third trimester having the lowest score, but it was significantly higher (p = 0.01) among those in the second trimester than those in their first and third trimesters in the physical health domain only (Table 5).

**Table 5:** Association between QoL and various domains with sociodemographic characteristics. QoL: quality of life

Variable	Overall QoL*	Physical health	Psychological	Social relations	Environment
Age (years)					
≤32	85.56 ± 9.24	66.29 ± 9.12	62.42 ± 8.92	72.97 ± 8.57	66.01 ± 7.74
>32	84.39 ± 10.05	63.18 ± 9.54	63.23 ± 9.69	72.52 ± 11.14	66.01 ± 9.29
P-value	0.28	<0.01	0.44	0.68	1.00
Educational status					
≤Secondary	82.97 ± 10.17	61.58 ± 8.25	59.63 ± 8.78	68.92 ± 9.97	62.63 ± 7.17
Tertiary	85.63 ± 9.38	65.87 ± 9.53	63.67 ± 9.21	73.85 ± 9.45	66.96 ± 8.54
P-value	0.04	<0.01	0.01	<0.01	<0.01
Religion					
Christianity	85.61 ± 8.86	65.27 ± 9.48	62.80 ± 9.47	73.08 ± 9.85	66.27 ± 8.75
Islam/Traditional	83.67 ± 11.15	64.07 ± 8.97	62.76 ± 8.75	72.02 ± 9.59	65.37 ± 7.65
P-value	0.13	0.27	0.97	0.36	0.35
Marital status					
Single/Divorced/Separated	82.35 ± 13	65.13 ± 8.04	59.56 ± 7.62	74.51 ± 13.65	66.54 ± 10.23
Married	85.19 ± 9.37	64.91 ± 9.50	62.96 ± 9.32	72.68 ± 9.54	65.98 ± 8.36
P-value	0.40	0.92	0.09	0.59	0.83
Ethnicity					
Yoruba	84.80 ± 9.54	64.50 ± 9.56	62.53 ± 9.21	72.85 ± 9.70	65.67 ± 8.18
Igbo	85.67 ± 9.80	66.61 ± 8.64	63.19 ± 9.27	72.92 ± 9.29	67.45 ± 9.56
Hausa/Others	90.00 ± 10.95	67.26 ± 10.21	70.14 ± 9.65	68.06 ± 17.01	66.67 ± 8.54
P-value	0.36	0.24	0.13	0.49	0.33
Occupation					
Waged employment	88.72 ± 9.97	66.26 ± 10.14	65.25 ± 10.18	74.56 ± 10.82	68.35 ± 9.54
Unemployed	84.17 ± 8.30	64.14 ± 9.45	60.24±9.75	72.57 ± 7.57	66.67 ± 7.75
Self-employed	83.49 ± 9.15	64.40 ± 9.09	62.00 ± 8.62	72.02 ± 9.47	64.93 ± 7.84
P-value	<0.01	0.26	0.01	0.11	<0.01
Accommodation					
Self-owned	83.57 ± 8.70	63.14 ± 8.59	62.50 ± 9.07	72.62 ± 14.14	67.75 ± 7.94
Rented	85.18 ± 9.69	65.08 ± 9.49	62.81±9.29	72.79 ± 9.30	65.85 ± 8.48
P-value	0.36	0.26	0.86	0.95	0.24
Parity					
0	85.70 ± 9.29	66.23 ± 9.07	63.61 ± 7.72	73.31 ± 7.94	66.73 ± 7.72
1	84.24 ± 9.46	64.89 ± 9.89	62.85 ± 10.34	73.09 ± 10.81	66.02 ± 8.92
2	85.20 ± 9.99	64.12 ± 8.82	62.43 ± 9.35	72.18 ± 9.52	66.12 ± 7.85
≥3	86.92 ± 9.47	65.12 ± 12.81	60.58 ± 6.50	72.44 ± 12.90	60.34 ± 12.20
P-value	0.63	0.49	0.67	0.84	0.90
Trimester (gestational age)					
First	87.14 ± 9.51	59.69 ± 11.24	59.52 ± 8.23	69.05 ± 9.27	62.50 ± 6.99
Second	85.66 ± 9.19	66.16 ± 9.28	62.36 ± 8.69	73.07 ± 8.99	65.64 ± 7.93
Third	83.81 ± 10.29	63.01 ± 9.28	63.74 ± 10.23	72.46 ± 11.08	66.87 ± 9.34
P-value	0.21	0.01	0.28	0.51	0.24

## Discussion

Medical disorders during pregnancy alter a woman’s lifestyle and may affect her psychologically or make her feel as though she has no control over her body [12]. Our study assessed the QoL of pregnant women with medical disorders and identified the factors associated with it. The overall QoL scores and health satisfaction were good despite the medical disorders in the study population and comparable with the findings of Wu et al. and Bien et al. [10,14]. This might be because most of our study participants had a higher level of education, earned more than the national monthly minimum wage ($18), and received multidisciplinary medical care. Higher education levels, good and early antenatal care, and high income have been associated with higher QoL in different studies [12,15]. Education plays a crucial role in health awareness, including the level of understanding of medical disorder(s), pregnancy needs and demands, and good health-seeking behavior [12,15]. People without formal education are usually unable to obtain desirable employment and have fewer chances of achieving financial success.

Education helps people better comprehend the world they live in and, as a result, perceive that they have the power to change it [16]. We found a significant association between educational status with the overall QoL across all the other domains. High scores in the QoL domains (physical health, psychological, social relations, and environment) were observed in women with tertiary education compared to those with secondary education or lower education. In the analysis of the non-financial advantages of education, Oreopoulos and Salvanes concluded that education is one of the most significant predictors of one’s health status [17]. Additionally, this study showed that employed women had higher QoL scores in the psychological and environmental domains and reported much better overall QoL.

Moreover, their good overall QoL might also be attributed to their access and utilization of antenatal care in a specialist health facility which availed them of comprehensive information about their medical condition, potential risks and complications, prompt medical care, available resources, and treatment options which would have contributed to their ability to make informed decisions and patient-centered care. These are all expected to cumulate into improved and good pregnancy outcomes [18]. The good overall QoL among pregnant women is in keeping with reports by Mazuchová et al. and Ishaq et al. in the Czech Republic and Pakistan, respectively [15,19].

The mean scores of QoL of those who had a single medical disorder were slightly higher than those with multiple medical disorders, though this was not statistically significant. The women’s QoL scores in each WHOQOL-BREF domain are comparable with findings from other studies. Iwanowicz-Palus et al. and Bien et al. reported that pregnant women with a medical disorder (hyperglycemia) had higher overall QoL scores [14,20].

The highest scores were found in the social domain which corroborated other studies on QoL in pregnant women with a medical disorder of hyperglycemia during their antenatal and postpartum periods [14,20,21]. A strong social support system, including family and friends, has been reported to relieve the physical and emotional burdens associated with medical illnesses during pregnancy [22]. On the other hand, the lowest scores were noted in the psychological QoL domain of the WHOQOL-BREF. Similar findings were also reported in several studies among pregnant women [21,23].

In general, the psychological domain involves feelings of acceptance, satisfaction, concentration, meaning of life, and psychological disorders which could have contributed to the reduced scores despite the social support. Low QoL in psychological scores is probably due to prenatal anxiety, depression, stress, and fear of childbirth [24]. This underscores the importance of a psychologist’s input and a multidisciplinary approach in the management of pregnant women. Furthermore, the high QoL scores in the physical health domain were significantly associated with younger age. Considering that younger pregnant women are often anticipated to be more physically active than older pregnant women, this outcome was not unexpected and supported by reports from previous studies. According to a study by Balikova and Buzgova, women 29 years and younger had higher QoL than older women [25]. Further, Mazuchova et al. reported the best QoL in younger women when compared with middle-aged women and the worst in the older age group [19].

Gestational age was the only obstetric characteristic significantly associated with women’s QoL in the physical health domain. Pregnant women in the second trimester perceived higher QoL in this domain than those in the third trimester. The reason for the lower QoL in the physical health domain in the third trimester may be attributed to a large uterus causing strain and discomfort with movement and usual activities, thus our findings could be due to some physical and mental changes during pregnancy [10]. Pregnant women in the first trimester had higher QoL scores in all domains compared to their counterparts in the third trimester. This finding was similar to that observed in studies in Morocco and France where pregnant women had lower QoL in the second and third trimesters [26,27].

In this study, pregnant women presented with diverse medical disorders ranging from gastrointestinal tract, cardiovascular, and respiratory, to psychiatric illnesses, though a single illness was more prevalent compared with multiple medical illnesses. The gastrointestinal system was most affected among pregnant women with medical disorders. Gastrointestinal disorders, mainly nausea and vomiting, gastroesophageal reflux, and constipation, represent some of the most frequent complaints during pregnancy probably due in part to elevated hormones such as progesterone, human chorionic gonadotropin, and/or prostaglandins, as well as increased gastrin production, and delayed gastric emptying [28]. Regarding gastrointestinal disorders in pregnancy, it is pertinent that the right approach, diagnosis, and treatment of pregnant women be considered by obstetricians in collaboration with gastroenterologists when required to improve the clinical outcome [28].

The occurrence of cardiovascular diseases, which comprised all hypertensive disorders, was also prevalent in this study. This finding is consistent with earlier rates reported by Babah et al. and Chaudhary et al. in India [29,30]. The hypertensive disorder still ranks as one of the top causes of maternal mortality globally.

Some women presented with gestational diabetes mellitus but in combination with illnesses such as asthma, hepatitis B, HIV, or hypertensive disorders. All these diseases have been linked to adverse pregnancy outcomes. Hematological disorders occurred in about 7% of the participants. Anemia is a well-known common hematological disorder in pregnancy in Nigeria, as noted in our study [31,32]. This study further investigated the association between the women’s characteristics and QoL. The factors associated with high QoL in women with medical illnesses across the different domains were younger age, high educational status, being employed, and gestational age.

This study is not without some limitations. The duration of onset of these medical illnesses was not factored into this study as well as the presence of complications that could affect the QoL of pregnant women. It was also difficult to assess which of the disorders in those with multiple medical disorders had more impact on QoL. A mixed-method study including a qualitative assessment of the QoL of the women in groups might provide more insights into the challenges they face in each domain and more information on their QoL. However, this study did not only assess the impact of one or two medical illnesses on the QoL of these pregnant women but also of multiple medical disorders in pregnancy and reported the overall QoL satisfaction rate across different gestational ages. All QoL domains were assessed adequately using the highly reliable WHOQOL-BREF tool which makes the study reliable and reproducible.

## Conclusions

The QoL of pregnant women with medical illnesses was good, highest in the social domain and lowest in the psychological domain. The factors significantly associated with their high QoL scores were age, educational status, employment status, and gestational age. There is a need to improve the provision of socioeconomic and psychological support for pregnant women with medical illnesses. Moreover, ensuring a girl child is educated might go a long way toward improving their QoL during pregnancy. Despite the positive QoL in this study, there is a need to provide more care and attention to pregnant women with medical illnesses by ensuring a multidisciplinary approach and a psychologist’s involvement in their treatment.

## Appendices

Questionnaire

Dear respondents,

The researcher is a consultant Obstetrician and Gynecologist specializing in Fetal-maternal Medicine. He is an associate lecturer in the Obstetrics and Gynecology Department, College of Medicine, University of Ibadan, conducting this study titled: Quality of Life of Pregnant Women With Medical Disorders in Ibadan, Nigeria. All information provided will be treated with confidentiality and used for the intended purpose.

Thank you for your time and cooperation.

Name: Dr. Oni O.O                                 Email address: olaoluoni43@yahoo.com

Below is a consent form that serves as a binding contract for confidentiality and voluntariness in participating in the research.

Participants’ consent form

I have read the description of the research. I understand that my participation is voluntary. I know enough about the purpose, methods, risks and benefits of the research study to judge that I want to take part in it. I understand that I may freely stop being part of this study at any time. I thereby give my consent to be part of the research as I was not coerced into partaking in the study.

Date:                                    Signature:                        ________

Section A: Sociodemographic and obstetric features

1.      Age: _______ in years

2.      Gestational age: ____________in weeks

Circle the correct option concerning you in the following questions:

3.     What is your level of education? a. None b. Primary school c. Secondary school d. College/University

4.      What is your religion?  a. Christianity b. Islam c. Traditional d. Others (Specify)                        

5.     What is your ethnicity?  a. Yoruba b. Igbo c. Hausa d. Others (Specify)6.      What is your marital status? a. Single 28b.   Married c. Separated/Divorced

7.      What is your occupation? a. Employed b. Unemployed   c. Business (Trading, Hairdressing, etc.)

8.      What is your accommodation like? a. Personal/owned b. Rented/Tenants

9.     What is your monthly Income? a. Less than 5,000 naira b. 5000-20,000 naira

     c. 20,000-50,000 naira d. More than 50,000 naira

10.  How many children do you have? a. None b. 1 c. 2 d. 3  d..4 or more

11.  Do you attend antenatal clinics? a. Yes b. No

12.  Have you been diagnosed with any health condition before or during pregnancy?

 a. Yes b. No

13.  If yes, what is your diagnosis? ______________________________________

14.  Has your visit to the clinic been more frequent due to your diagnosis?

a. Yes b. No

15.  Have you spent more money on your antenatal care than you anticipated since you had been diagnosed?  a. Yes b. No

16.  Have you been admitted to the ward due to your health condition in this pregnancy? 

a. Yes   b. No

17.  Was your diet affected due to your diagnosis?  a. Yes b. No

18.  Did you have the cause to take some days off work more than before because you were diagnosed with a health condition?  a. Yes b. No

19.  Have you been regular with your medications? a. Yes b. No

20.  Are the side effects tolerable? a. Yes b. No

21.  Describe how you feel about your current diagnosis during pregnancy.

____________________________________________________________________________________________________________________________

Section B: WHOQOL-BREF

Instructions: The following questions ask how you feel about your quality of life, health, or other areas of your life in the last four weeks. Circle the answer that appears most appropriate.

22. How would you rate your quality of life?   a. Very poor b. Poor c. Neither poor nor good d. Good e. Very good.

23. How satisfied are you with your health?   a. Very dissatisfied b. Dissatisfied c. Neither satisfied nor dissatisfied d. Satisfied e. Very satisfied.

The following questions ask about how much you have experienced certain things in the last four weeks. Circle the answer that appears most appropriate.

24. To what extent do you feel that physical pain prevents you from doing what you need to do?  a. Not at all   b. A little c. A moderate amount d. Very much e. An extreme amount.

25. How much do you need any medical treatment to function in your daily life?  a.  Not at all   b. A little c. A moderate amount d. Very much e. An extreme amount. 

26. How much do you enjoy life? a. Not at all b. A little c. A moderate amount d. Very much e. An extreme amount. 

27. To what extent do you feel your life to be meaningful? a.  Not at all   b. A little c. A moderate amount d. Very much e. An extreme amount.  

28. How well are you able to concentrate?   a.  Not at all   b. A little c. A moderate amount d. Very much e. An extreme amount.  

29. How safe do you feel in your daily life?   a.  Not at all   b. A little c. A moderate amount d. Very much e. An extreme amount.  

30. How healthy is your physical environment?  a.  Not at all   b. A little c. A moderate amount d. Very much e. An extreme amount.  

The following questions ask about how completely you experienced or were able to do certain things in the last four weeks. Circle the answer that appears most appropriate.

31. Do you have enough energy for everyday life?  a.  Not at all   b. A little c. A moderate amount d. Very much e. An extreme amount.  

32. Are you able to accept your bodily appearance?  a.  Not at all   b. A little c. A moderate amount d. Very much e. An extreme amount.  

33. Have you enough money to meet your needs?   a.  Not at all   b. A little c. A moderate amount d. Very much e. An extreme amount.  

34. How available to you is the information that you need in your day-to-day life?  a.  Not at all   b. A little c. A moderate amount d. Very much e. An extreme amount.  

35. To what extent do you have the opportunity for leisure activities?  a.  Not at all   b. A little c. A moderate amount d. Very much e. An extreme amount.  

36. How well are you able to get around? a. Very poor b. Poor c. Neither poor nor good d. Good e. Very good. 

37. How satisfied are you with your sleep?  a.  Very dissatisfied b. Dissatisfied c. Neither satisfied nor dissatisfied d. Satisfied e. Very satisfied. 

38. How satisfied are you with your ability to perform your daily living activities?  a. Very dissatisfied b. Dissatisfied c. Neither satisfied nor dissatisfied d. Satisfied e. Very satisfied. 

39. How satisfied are you with your work capacity?  a.  Very dissatisfied b. Dissatisfied c. Neither satisfied nor dissatisfied d. Satisfied e. Very satisfied. 

40. How satisfied are you with yourself?  a.  Very dissatisfied b. Dissatisfied c. Neither satisfied nor dissatisfied d. Satisfied e. Very satisfied. 

41. How satisfied are you with your personal relationships?  

42. How satisfied are you with your sex life?  a. Very dissatisfied b. Dissatisfied c. Neither satisfied nor dissatisfied d. Satisfied e. Very satisfied. 

43. How satisfied are you with the support you get from your friends?  a. Very dissatisfied b. Dissatisfied c. Neither satisfied nor dissatisfied d. Satisfied e. Very satisfied. 

44. How satisfied are you with the conditions of your living place?  a. Very dissatisfied b. Dissatisfied c. Neither satisfied nor dissatisfied d. Satisfied e. Very satisfied. 

45. How satisfied are you with your access to health services?  a. Very dissatisfied b. Dissatisfied c. Neither satisfied nor dissatisfied d. Satisfied e. Very satisfied. 

46. How satisfied are you with your transport?   a. Very dissatisfied b. Dissatisfied c. Neither satisfied nor dissatisfied d. Satisfied e. Very satisfied.  

47. How often do you have negative feelings such as blue mood, despair, anxiety, and depression?  a. Never b. Seldom c. Quite often d. Very often e. Always
